# Parkinson’s disease-associated mutations in DJ-1 modulate its dimerization in living cells

**DOI:** 10.1007/s00109-012-0976-y

**Published:** 2012-11-27

**Authors:** Mariaelena Repici, Kornelis R. Straatman, Nadia Balduccio, Francisco J. Enguita, Tiago F. Outeiro, Flaviano Giorgini

**Affiliations:** 1 Department of Genetics, University of Leicester, Leicester, UK; 2 Centre for Core Biotechnology Services, University of Leicester, Leicester, UK; 3 Unidade de Biologia Celular, Instituto de Medicina Molecular, Lisboa, Portugal; 4 Cell and Molecular Neuroscience Unit, Instituto de Medicina Molecular, Lisbon, Portugal; 5 Instituto de Fisiologia, Faculdade de Medicina de Lisboa, Lisboa, Portugal; 6 Department of Neurodegeneration and Restorative Research, University Medizin Göttingen, Göttingen, Germany

**Keywords:** Parkinson’s disease, DJ-1, Dimerization, Bimolecular fluorescence complementation

## Abstract

Mutations in the protein DJ-1 cause recessive forms of early onset familial Parkinson’s disease (PD). To date, most of the causative mutations studied destabilize formation of DJ-1 homodimers, which appears to be closely linked to its normal function in oxidative stress and other cellular processes. Despite the importance of understanding the dimerization dynamics of this protein, this aspect of DJ-1 biology has not previously been directly studied in living cells. Here, we use bimolecular fluorescence complementation to study DJ-1 dimerization and find not only that DJ-1 forms homodimers in living cells but that most PD causative DJ-1 mutations disrupt this process, including the L166P, M26I, L10P, and P158∆ mutations. Interestingly, the E64D mutant form of DJ-1 retains the ability to form homodimers. However, while wild-type DJ-1 dimers are stabilized under oxidative stress conditions, we find that the E64D mutation blocks this stabilization. Furthermore, our data show that the E64D mutation potentiates the formation of aggresomes containing DJ-1. We also observe that while the widely studied L166P mutation prevents DJ-1 from forming homodimers or heterodimers with wild-type protein, the mutant protein is able to partially disrupt formation of wild-type homodimers. In summary, by investigating DJ-1 dimerization in living cells, we have uncovered several novel properties of PD causative mutations in DJ-1, which may ultimately provide novel insight into PD pathogenesis and possible therapeutic options.

**Electronic supplementary material:**

The online version of this article (doi:10.1007/s00109-012-0976-y) contains supplementary material, which is available to authorized users.

## Introduction

Parkinson’s disease (PD) is the second most common neurodegenerative disorder after Alzheimer’s disease. However, the mechanisms underlying causation and progression of this disorder are not well understood. While sporadic PD accounts for more than 90 % of all cases, the study of rare genetic forms may contribute to our understanding of the cellular mechanisms underlying the pathogenesis of both idiopathic and familial forms of the disease. Mutations in the *PARK7* gene account for ~1–2 % of the sporadic cases of early onset recessive PD [1]. Since 2003, when a large homozygous deletion and a homozygous missense mutation in the *PARK7* gene were first reported in two European families, numerous other mutations have been identified [2]. Among these, homozygous and compound heterozygous mutations are clearly associated with early onset PD, while it is unclear if heterozygous mutations are PD causative [3].

*PARK7*-related PD symptomatology is characterized by levodopa-responsive parkinsonism with an early age at onset and generally no atypical signs. *PARK7* encodes for DJ-1, a small conserved protein of 189 amino acids (aa), which is not only ubiquitously expressed and primarily localized to the cytoplasm but also found in the nucleus and associated with mitochondria [4–7]. Structural studies have shown that the monomeric form of DJ-1 contains a conserved α/β sandwich fold found in members of the ThiJ/PfpI protein superfamily [8, 9] and that, at least in vitro, DJ-1 exists as homodimer, which appears to be critical for its normal physiological function [10, 11].

DJ-1 has been implicated in several pathways associated with PD pathogenesis, but the exact molecular mechanisms underlying its contribution to disease are still elusive. Nonetheless, it is clear that this protein plays an important role in cellular response to oxidative stress and is required for mitochondrial health [12, 13]. Despite the rare incidence of DJ-1 mutations in PD, the study of DJ-1 biology can provide important clues to altered cellular pathways in PD. Thus, understanding how the causative DJ-1 mutations interfere with the structure, function, and localization of DJ-1 protein is of critical importance.

The L166P mutation [5] severely perturbs DJ-1 protein structure, resulting in the formation of a spontaneously unfolded protein [14]. Furthermore, using biochemical approaches, it was found that the L166P mutant protein does not dimerize [8, 14] and is extremely unstable when expressed in mammalian cell lines [14–18]. In comparison, little is known about the effect of other DJ-1 mutations on its structure/function. The expression levels of the M26I mutant are decreased in cell lines, though to a lesser degree than the L166P mutant, and the M26I protein may retain the ability to dimerize [4, 19]. However, the M26I homodimer is less stable than the wild-type dimer [20]. Two additional causative DJ-1 mutations—L10P and P158Δ—are characterized by decreased stability and impaired homodimer formation [21]. Interestingly, the crystal structure of the E64D mutant protein is not altered [22], and this mutant protein is stable in cells and can dimerize in manner similar to WT DJ-1 [15, 20]. Thus, the studies to date shed little light on how the E64D mutation is causative in PD and suggest a functional divergence in the nature of the disease-causing DJ-1 mutations.

Here, we take advantage of bimolecular fluorescence complementation (BiFC) to elucidate DJ-1 function in living cells and study a panel of DJ-1 mutations (L166P, E64D, M26I, L10P, and P158Δ). To date, only biochemical approaches have been used to analyze DJ-1 dimerization, providing little insight into the dynamics of this process in cells. Importantly, we demonstrate that BiFC is a powerful tool for the study of DJ-1 dimerization in living cells. We also find that—uniquely among the mutant proteins studied—the E64D mutation does not impair dimer formation in normal conditions but does alter dimerization dynamics under oxidative stress conditions. Furthermore, we find that the E64D dimers show an increased propensity to form aggresomes in living cells, which may have implications for its role in pathogenesis. In summary, our study finds that dimerization of DJ-1 in living cells is an exquisitely sensitive process and uncovers novel aspects of the role causative mutations play in DJ-1 dysfunction, which may open novel avenues for therapeutic intervention in PD.

## Materials and methods

### Generation of DJ-1 constructs

The two WT DJ-1 BiFC constructs (DJ1-GN173 and DJ1-CC155) were generated by PCR-based cloning into pcDNA3.1 vector. The constructs encoding five mutant forms of DJ-1 (L166P, M26I, P158Δ, L10P, and E64D) were produced via a combination of the Stratagene Quikchange II XL and Phusion Site-Directed Mutagenesis (F-541, Finnzymes) methods, using both WT DJ1-GN173 and WT DJ1-CC155 as templates. The primer pairs used are listed in Table S1. All constructs were verified by DNA sequencing.

### Cell culture and transient transfection techniques

HEK 293T cells were plated on 35-mm ibiTreat dishes (IBIDI) at the density of 1 × 10^5^ cells/dish for confocal laser scanning microscope (CLSM) analysis in living cells, or in six-well plates (1.5 × 10^5^ cells/well) precoated with 0.01 % poly-l-lysine solution for BiFC experiments, and cultured in Dulbecco’s modified Eagle’s medium, high glucose, supplemented with 10 % fetal bovine serum, 100 U/ml penicillin, and 100 μg/ml streptomycin, at 37 °C in a 95 % air/5 % CO^2^ atmosphere. Cells were plated on coverslips precoated with 0.01 % poly-l-lysine solution for immunocytochemistry studies. Transfection was performed 24 h after plating using the Effectene Transfection Reagent kit (Qiagen) using procedures supplied by the manufacturer. The percentage transfection efficiency was routinely >50 %. Cells were lysed either 24 or 48 h after transfection for immunoblotting studies. For oxidative stress treatments, cells were exposed 24 h after transfection to either paraquat 200 μM for 24 h or to hydrogen peroxide 1 mM for 2 h. After the treatment, cells were washed with phosphate-buffered saline (PBS), and fresh medium was added before BiFC analysis.

### Fluorescence complementation assay and live cell imaging

Cells were imaged 24 or 48 h posttransfection on an Olympus ScanˆR screening station equipped with a 20× LUCPlanFLN objective (NA = 0.45) and a Hamamatsu ORCA-AG CCD camera. The light source was a MT-20 illumination system (Olympus Biosystems) with a high-stability 150 W xenon arc burner. During imaging, the cells were kept at 37 °C and 5 % CO_2_. Green fluorescent protein (GFP) BiFC fluorescence was detected using a 492/18 nm excitation filter and 535/50 nm emission filter. To detect transfected cells and to compare the efficiencies of fluorescence complementation between wild-type and mutant DJ-1 proteins, HEK 293T cells were cotransfected with the pcDNA3.1 plasmid-expressing red fluorescent protein (RFP). RFP expression was imaged using excitation at 556/30 nm and emission between 590 and 650 nm using the filterset 51019 (Chroma). The ratio between GFP and RFP emissions was quantified for every cell expressing RFP after subtraction of the background signal, 100 pictures were taken per well in each experiment. Typically, several hundred cells were identified and quantified for each condition in each independent experiment and analyzed using the ScanˆR analysis software.

### Immunocytochemistry and confocal laser scanner microscopy analysis

Twenty-four or 48 h after transfection, cells were fixed in 4 % paraformaldheyde in PBS for 20 min at 37 °C and then incubated in 1 % bovine serum albumin (BSA) in PBS 0.2 % Triton for 30 min at room temperature. Primary antibodies were diluted 1:200 (anti-DJ-1, sc-27006, Santa Cruz Biotechnology), 1:100 (anti-DJ-1, sc-55572, Santa Cruz Biotechnology), 1:1,000 (anti GFP, ab6556 Abcam), 1:200 (anti-HtrA2, AF1458, R&D Systems), 1:100 (anti-Vimentin, 5741, Cell Signalling Technology), in blocking solution and incubated overnight at 4 °C. After washing in PBS, cells were incubated for 2 min in 1:2,000 Hoechst 33342 trihydrocloride, 10 mg/ml solution (Invitrogen), in PBS. Secondary antibodies were diluted 1:500 (Alexa Fluor 546 antigoat and anti rabbit) in PBS 0.2 % Triton + 1 % BSA and incubated at room temperature for 1 h. Finally, cells were rinsed in PBS, and coverslips were mounted in Mowiol.

CLSM analysis was performed using an Olympus FV1000 confocal laser scanning microscope. Cells were imaged in sequential mode using a 60× UPlanSAPO Olympus objective, Kalman filter of 4, and a zoom of 1.5. The following settings were used: for Hoechst, excitation of 405 nm laser line, emission detected between 425 and 475 nm; for GFP BiFC, excitation of 488 nm laser line, emission detected between 500 and 545 nm; for RFP/Alexa 546, excitation of 559 nm laser line, emission 575–675 nm; and for Alexa 647, excitation of 635 nm laser line and emission of 655–755 nm.

### Immunoblotting

Cells were washed twice with sterile PBS and then lysed on ice for 10 min in lysis buffer [23]. Lysates were centrifuged at 13,000 rpm for 10 min at 4 °C. Supernatants were collected, and protein concentration was determined by the Bradford method. Samples were stored at−80 °C until used. Proteins were separated on a 10 % SDS polyacrylamide gel (10 μg of total proteins per well) and transferred to a polyvinylidene difluoride membrane. Membranes were incubated for 1 h in TBST 5 % milk to saturate all non-specific binding sites (blocking solution). Incubation with primary antibodies was overnight at 4 °C, using goat anti-DJ-1 antibody (1:2,000; sc-27006, Santa Cruz Biotechnology) or mouse anti-tubulin (1:1,000; sc-8035, Santa Cruz Biotechnology). Blots were developed using horseradish peroxidase-conjugated secondary antibodies (1:10,000; PI-9500 horse antigoat Vector Laboratories and 1:80,000; 31430 goat antimouse Thermo Scientific Pierce) and the ECL chemiluminescence system (SuperSignal West Dura Extended Duration Substrate, Thermo Scientific).

### Aggresome quantification

HEK 293T cells were transfected with either the two WT DJ-1 or the two E64D DJ-1 BiFC constructs, fixed 48 h after transfection, subjected to Hoechst 33342 trihydrocloride staining and analyzed for aggresome formation. An aggresome was defined as a single, BiFC positive, perinuclear inclusion. For each experiment, 50 pictures per well were taken randomly, and DAPI-positive cells were counted and evaluated for the presence of aggresomes in a blinded manner. Experiments were repeated three times, and an unpaired, two-tailed *t* test was used to assess statistical significance (data = mean ± SEM; *P* = 0.0018).

### Molecular dynamics simulations and structural analysis

Molecular dynamics simulations in solvated media were performed with the GROMACS 4 package using the all-atom GROMOS96 forcefield. Coordinates of the DJ-1 wild-type proteins for the simulations were obtained from the PDB database (PDB codes: 1PF5 for the DJ-1 monomer, and 1SOA for the dimer). Mutants were manually built by replacing the selected residues in the coordinates using Coot software [24]. All the simulations were started with the native conformations of the peptides with a protonation of side chains consistent with a pH = 7. Proteins were solvated in a water box of 100 × 100 × 100 Å and a density of 1 g/cm^3^. The solvated models were energy minimized by conjugated gradient for 1,000 steps to eliminate steric clashes between atoms. All the systems were equilibrated by simulated annealing with slow temperature decreasing from 2,500 to 300 K over 1,000 cycles. Molecular dynamics simulations were then performed over 1,000 ps at 300 K and data collected every 1 ps. All the molecular representations depicted in the figures were built using Pymol. Calculation of continuous molecular surface properties was performed using VASCo software [25]. Polar patches on the protein surfaces were located by using HotPatch software [26].

### Statistical analysis

BiFC data were analyzed with Prism 5 (GraphPad), using the Kruskal–Wallis test followed by post hoc analysis with a Dunn test. *P* < 0.05 is considered significant for any set of data. In all experiments, results are expressed as means ± SEM.

## Results

### The L166P mutation prevents dimerization of DJ-1 in living human cells

**Fig. 1:**
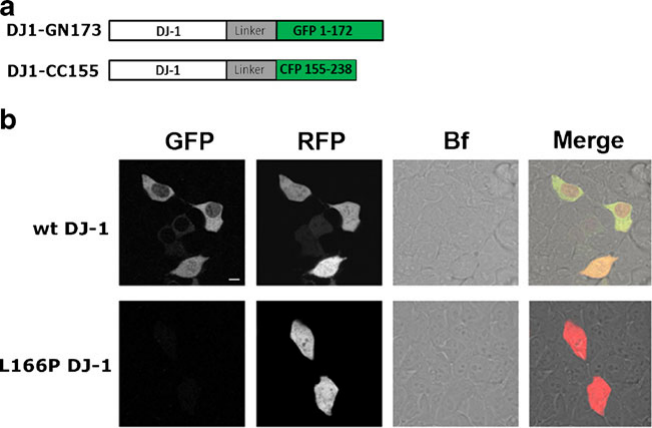
DJ-1 forms dimers in living cells. **a** Schematic representation of the BiFC constructs used. DJ-1-GN173 contains a polylinker region linking the 172 N-terminal amino acid residues of the GFP attached to the C terminus of full-length DJ-1. DJ-1-CC155 contains the 155–238 C-terminal residues of CFP linked via a polylinker region to the C terminus of full-length DJ-1. **b** One optical section taken on a confocal laser scanning microscope showing the complementation reaction driven by DJ-1 dimerization in living HEK293T cells. Both cytoplasmic and nuclear signal were observed in cells transfected with WT DJ-1 BiFC constructs. The L166P mutation completely prevents DJ-1 dimerization. HEK 293T cells were cotransfected with the pcDNA3.1 plasmid expressing RFP to allow for normalization of the BiFC signal. Scale bar = 10 μm

The ability of DJ-1 to form dimers in living cells was investigated using BiFC, which utilizes the reconstitution of nonfluorescent fragments of GFP to study protein–protein interactions [27]. We hypothesized that if DJ-1 dimerizes in living cells the two nonfluorescent fragments would be brought together, reassociate, and refold into a fluorescent complex. To explore this possibility, we generated two wild-type (WT) DJ-1 BiFC constructs (DJ-1-GN173 and DJ-1-CC155) predicted to generate a fluorescent complex upon DJ-1 dimerization (Fig. 1a). DJ-1-GN173 contains the N-terminal 173 amino acid (aa) residues of GFP connected by a polylinker to the C terminus of full-length DJ-1. On the other hand, DJ-1-CC155 comprises a C-terminal fragment of CFP (155–238 aa) fused to the C terminus of full-length DJ-1 via a polylinker region. We used CFP to complement GFP, as this combination allows for a better fluorescent complementation signal compared to the one obtained with the C terminus of GFP [28]. These constructs were transfected into HEK 293T cells individually or together and incubated for 24 h at either 30 or 37 °C—the lower temperature allowing for better maturation of the fluorophore [29]. No fluorescent signal was detected when cells were transfected with only one WT DJ-1 BiFC construct at either temperature (Fig. S1a), whereas the cotransfection of both constructs resulted in the formation of fluorescent signal (Fig. 1b) that was enhanced by incubation at 30 °C (Fig. S1b). Analysis of optical sections obtained using a CLSM revealed both cytoplasmic and nuclear localization of DJ-1. Thus, our experiments in living cells indicate that DJ-1 dimerizes, validating previous biochemical observations.

**Fig. 2:**
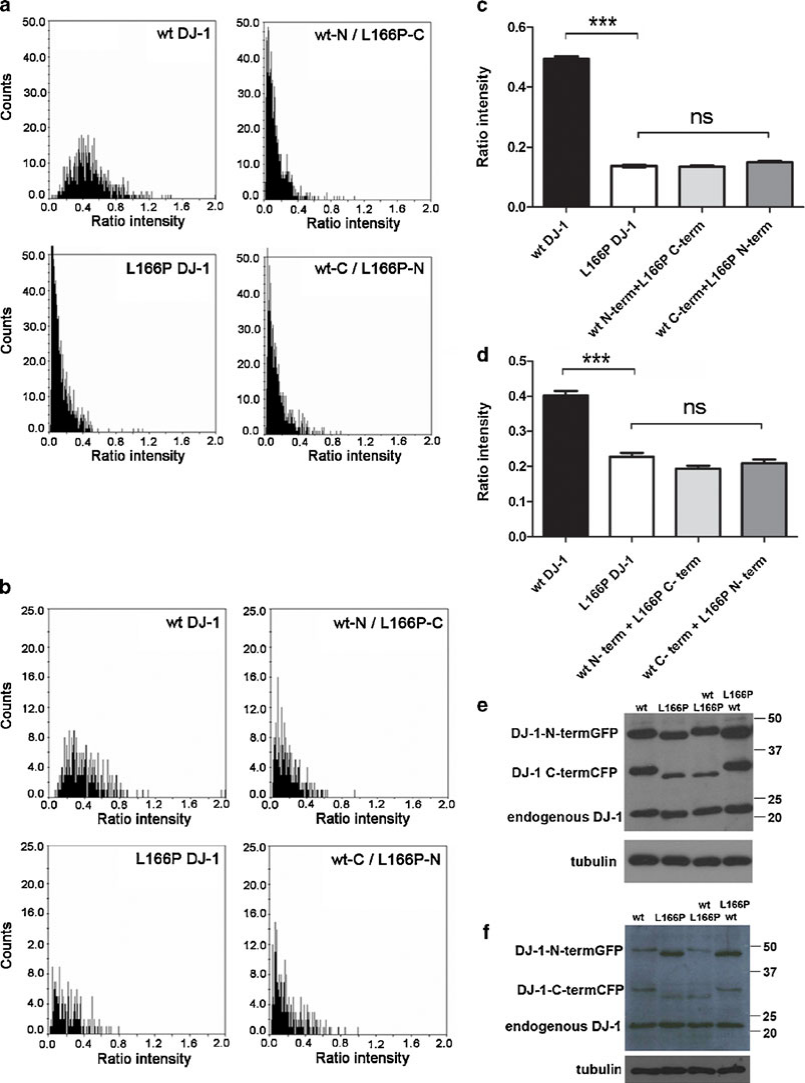
Effect of the L166P mutation on the efficiency of fluorescence complementation between DJ-1 BiFC constructs 24 h after transfection in HEK 293T cells. The distribution of ratios between GFP and RFP emissions in individual cells cotransfected with plasmids encoding the proteins is indicated in each graph (0.16 μg each) and plasmid encoding RFP (0.08 μg) is shown **a**. Emission intensities were corrected for background fluorescence using the ScanˆR analysis software. The histogram (**c**) shows the average ratio intensity (green/red) per well: No difference was observed in the complementation signal between the two L166P DJ-1 BiFC contructs and both combinations of WT DJ-1 and L166P mutant DJ-1 GFP halves, indicating that L166P DJ-1 is not able to dimerize with WT DJ-1. Data are expressed as mean ± SEM. **e** Anti DJ-1 immunoblots of the lysates obtained from transfected cell populations used in this BiFC experiment show the expression levels of the different BiFC constructs, compared to endogenous DJ-1 and to the loading control. In each lane, the upper band corresponds to DJ-1-GN173 and the intermediate band corresponds to DJ-1-CC155. **b** Effect of L166P mutation on the efficiency of fluorescence complementation between DJ-1 constructs, when BiFC constructs are normalized for protein levels. The amount of plasmids encoding the BiFC constructs used was: 0.1 μg for the two WT DJ-1 constructs; 0.21 μg for the two L166P DJ-1 constructs, and 0.1 μg WT DJ-1 + 0.21 μg L166P DJ-1 for both combinations of WT DJ-1 and L166P mutant DJ-1 GFP halves; 0.08 μg of the plasmid encoding RFP were used in each condition. **d** Average ratio intensity per well of the BiFC experiment shown in **b**. **f** Immunoblots of the lysates obtained from the transfected cell populations used in **b**. ***P < 0.001

To further validate BiFC as a system for the study of DJ-1 dimerization, we next tested DJ-1 carrying the L166P mutation, which has been shown to completely abrogate function of this protein [16, 17]. When we analyzed the signal obtained from expression of the two L166P DJ-1 BiFC constructs, we found that the L166P mutation virtually eliminated fluorescence complementation in HEK 293T cells (Fig. 1b). To quantify the effects of the L166P mutation on dimerization efficiency, we normalized the GFP emissions by the emissions of RFP produced from a cotransfected expression plasmid (see “Materials and methods”) (Fig. 2a, c). Supporting and extending previous biochemical analyses, we found that L166P strongly reduced BiFC signal, clearly indicating that this mutation prevents DJ-1 dimerization in living cells.

Immunoblotting analysis using the DJ-1 antibody revealed the presence of exogenous proteins with the expected molecular weights (DJ1-GN173 at ~45 kDa; and DJ1-CC155 at ~33 kDa), as well as endogenous DJ-1 (Fig. 2e). We found that expression levels of the WT DJ-1 BiFC constructs were comparable to endogenous DJ-1 levels and that the L166P mutation caused a decrease in the expression levels of the BiFC constructs compared to the WT DJ-1 BiFC constructs. To ascertain if the observed effect on fluorescence was entirely dependent on protein levels and not dimerization per se, we normalized L166P protein levels by transfecting the cells with increased DNA concentrations of the BiFC constructs (Fig. 2f and data not shown). BiFC experiments repeated under these conditions showed that, in addition to reducing protein levels, the L166P mutation also directly reduces the ability of DJ-1 to dimerize (Fig. 2b, d). Indeed, we observed no L166P DJ-1 fluorescence complementation signal despite an increased level of L166P DJ-1-GN173 protein relative to WT (Fig. 2f).

To gain insight into DJ-1 dimerization for individuals heterozygous for the L166P DJ-1 mutation, we next used BiFC to test the dimerization capacity of DJ-1 heterodimers. HEK 293T cells were transfected with both combinations of WT DJ-1 and L166P mutant DJ-1 GFP halves. A very low fluorescence ratio, comparable to the one obtained with the two L166P-DJ-1 constructs, was observed for both conditions, suggesting that L166P DJ-1 is not able to significantly dimerize with WT DJ-1 (Fig. 2c, d).

**Fig. 3:**
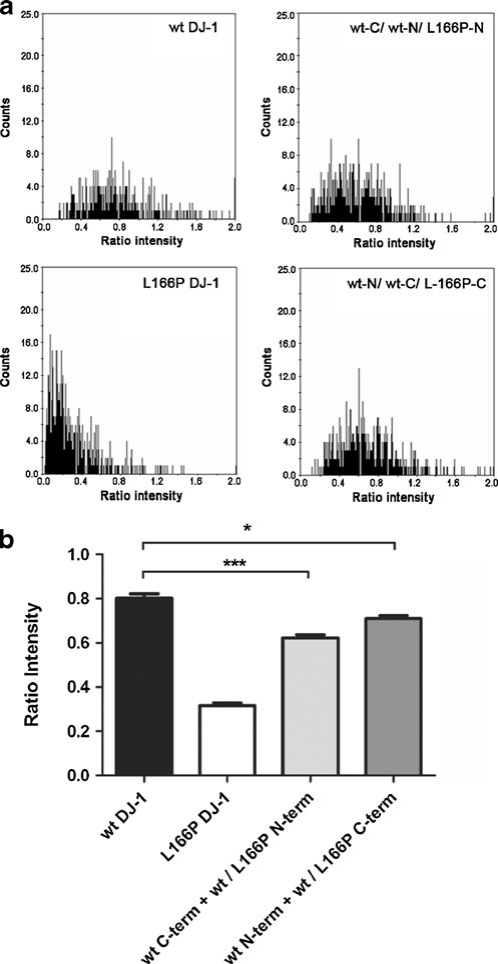
L166P DJ-1 disrupts formation of WT DJ-1 dimers. **a** Distribution of ratios between GFP and RFP emissions in individual cells. 0.16 μg of each of the DJ-1 BiFC constructs and 0.08 μg of the RFP encoding plasmid were used. **b** Average ratio intensity (green/red) per well. *P < 0.05; ***P < 0.001

As heterozygous individuals are predicted to still exhibit dimers of WT DJ-1, we chose to explore the potential of L166P DJ-1 to disrupt such dimers via competition experiments. We first determined that we could competitively reduce the amount of fluorescent signal by introducing a “cold” nontagged DJ-1 construct (Fig. S2). Having validated this approach, we then investigated whether the L166P mutant could affect WT DJ-1 dimerization efficiency. Cells were transfected with both WT DJ-1 constructs along with either the L166P DJ1-GN173 or the L166P DJ1-CC155 construct, thus creating competition for WT homodimer formation by L166P DJ-1. A significant reduction in fluorescence complementation was observed with either L166P construct (Fig. 3a, b), indicating that less dimer forms in the presence of this DJ-1 mutant protein. This suggests for the first time that, in addition to causing loss of normal DJ-1 function, the L166P mutation may generate a mutant form of DJ-1, which exhibits a negative effect on WT DJ-1 present in the cell.

### The E64D mutant does not abrogate DJ-1 dimerization in living cells

**Fig. 4:**
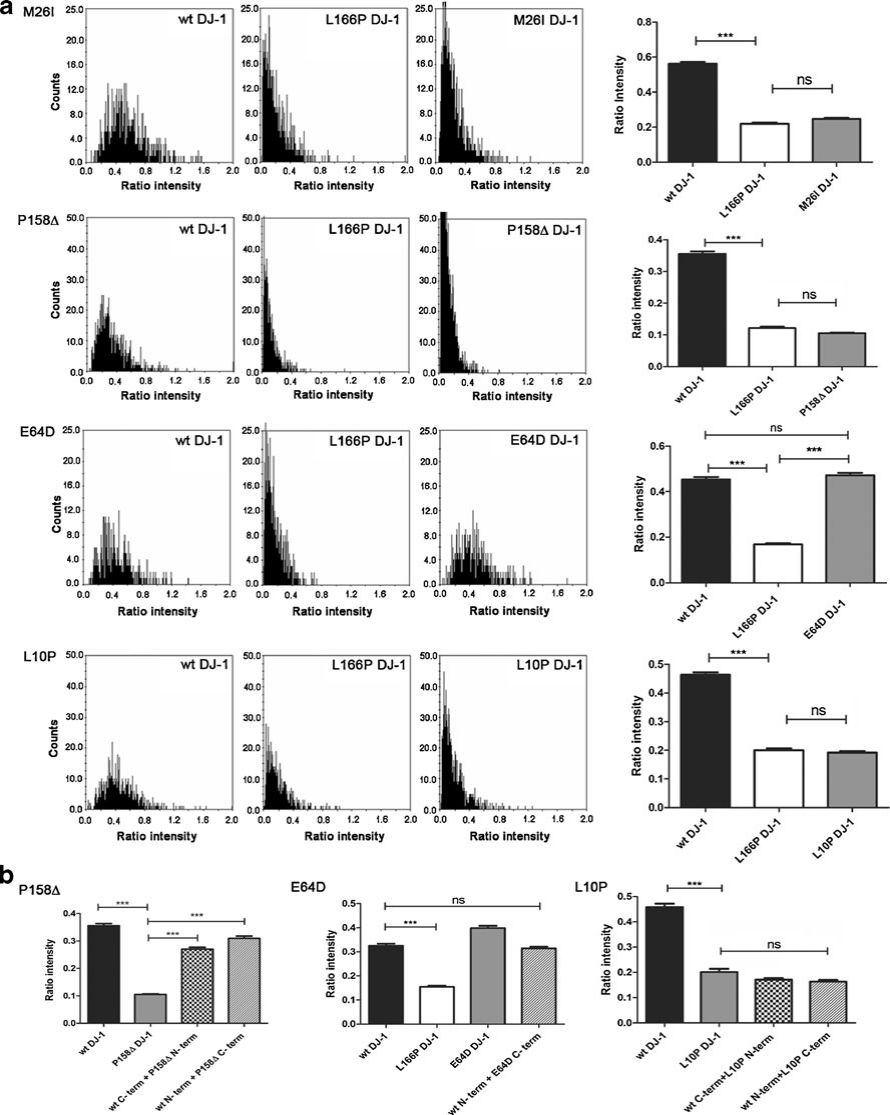
Effect of M26I, P158Δ, L10P and E64D mutations on the efficiency of fluorescence complementation between DJ-1 BiFC constructs. **a** BiFC signal under “homozygous conditions” for M26I, P158Δ, L10P, and E64D DJ-1 mutants. The distribution of ratios between GFP and RFP emissions in individual cells co-transfected with plasmids encoding the proteins indicated in each graph (0.16 μg each) and plasmid encoding RFP (0.08 μg) is shown. Emission intensities were corrected for background fluorescence. The histogram shows the average ratio intensity (green/red) per well for each experiment. ***P < 0.001. **b** BiFC signal under “heterozygous conditions” for the P158Δ, E64D, and L10P DJ-1 mutants. While the P158Δ mutant dimerizes with the WT protein at a reduced level and the L10P mutant is not able to dimerize with WT DJ-1, the E64D mutant completely retains its dimerization ability (no significant difference compared to the WT DJ-1 BiFC signal). ***P < 0.001

Next, we generated E64D, M26I, L10P, and P158Δ mutant versions of the DJ-1 BiFC constructs by site-directed mutagenesis and used the BiFC assay to investigate dimerization dynamics of these mutants in living cells. Cells expressing the M26I, L10P, or P158Δ mutant BiFC constructs exhibited a very low complementation signal—not significantly different than the L166P mutant—indicating that these DJ-1 mutants are unable to form dimers in living cells (Fig. 4a). Furthermore, immunoblotting analysis experiments detected a lower level of these mutant proteins compared to WT DJ-1, suggesting that the mutant proteins are rapidly degraded in living cells (data not shown). On the other hand, when HEK 293T cells were transfected with E64D mutant DJ-1 GFP halves, a fluorescence ratio comparable to the one obtained with the WT DJ-1 constructs was observed, indicating that E64D DJ-1 is able to dimerize in living cells (Fig. 4a). The level of E64D DJ-1 protein was comparable to WT DJ-1 control constructs, and analysis by CLSM showed a similar cytoplasmic and nuclear localization pattern as WT DJ-1 constructs (data not shown). Finally, we tested the ability of the L10P, P158∆, and E64D mutants to form heterodimers with WT DJ-1. Interestingly, while L10P DJ-1 is not able to dimerize with the WT protein, an intermediate level of complementation was obtained for the P158∆/WT DJ-1 heterodimer (Fig. 4b). On the other hand, no change in the BiFC signal was observed for E64D/WT DJ-1 heterodimers (Fig. 4b), further underscoring that E64D DJ-1 does not exhibit impaired dimerization under normal conditions.

### WT DJ-1 dimers, but not E64D DJ-1 dimers, are stabilized under oxidative stress conditions

**Fig. 5:**
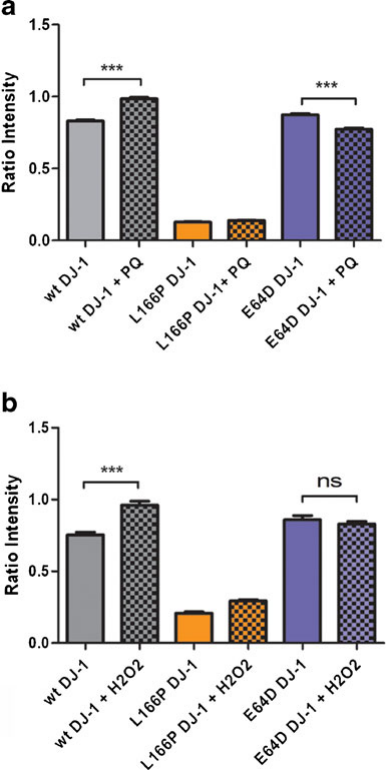
Oxidative stress stabilizes WT DJ-1 dimerization. HEK 293T cells were transfected with either two WT, L166P, or E64D DJ-1 BiFC constructs (0.16 μg of each BiFC plasmid and 0.08 μg of the RFP encoding plasmid) for 24 h and then subjected to oxidative stress by exposure to either **a** 200 μM paraquat for 24 h or **b** 1 mM hydrogen peroxide for 2 h. BiFC imaging was performed in normal medium immediately after the oxidative stress treatment. The histogram shows the average ratio intensity (green/red) per well ± SEM. ***P < 0.001

As DJ-1 plays an important role in cellular protection from oxidative stress [6, 30, 31], we next used BiFC to test the effect of oxidant treatments on the dimerization of WT and E64D DJ-1. We found that treatment with either paraquat or hydrogen peroxide increased BiFC fluorescence for WT DJ-1 (Fig. 5), indicating a stabilization of dimerization under oxidative stress conditions. Interestingly, we did not observe this increase with E64D DJ-1 BiFC signal. Indeed, the fluorescent signal for the E64D DJ-1 dimer was decreased in the case of the paraquat treatment (Fig. 5a) and unchanged with hydrogen peroxide treatment (Fig. 5b). These data suggest that, though E64D mutant retains its dimerization ability, it does not behave like WT DJ-1 under oxidative stress conditions, which may have implications for its role in PD pathogenesis.

### E64D DJ-1 dimers form cytoplasmic inclusions in living cells

**Fig. 6:**
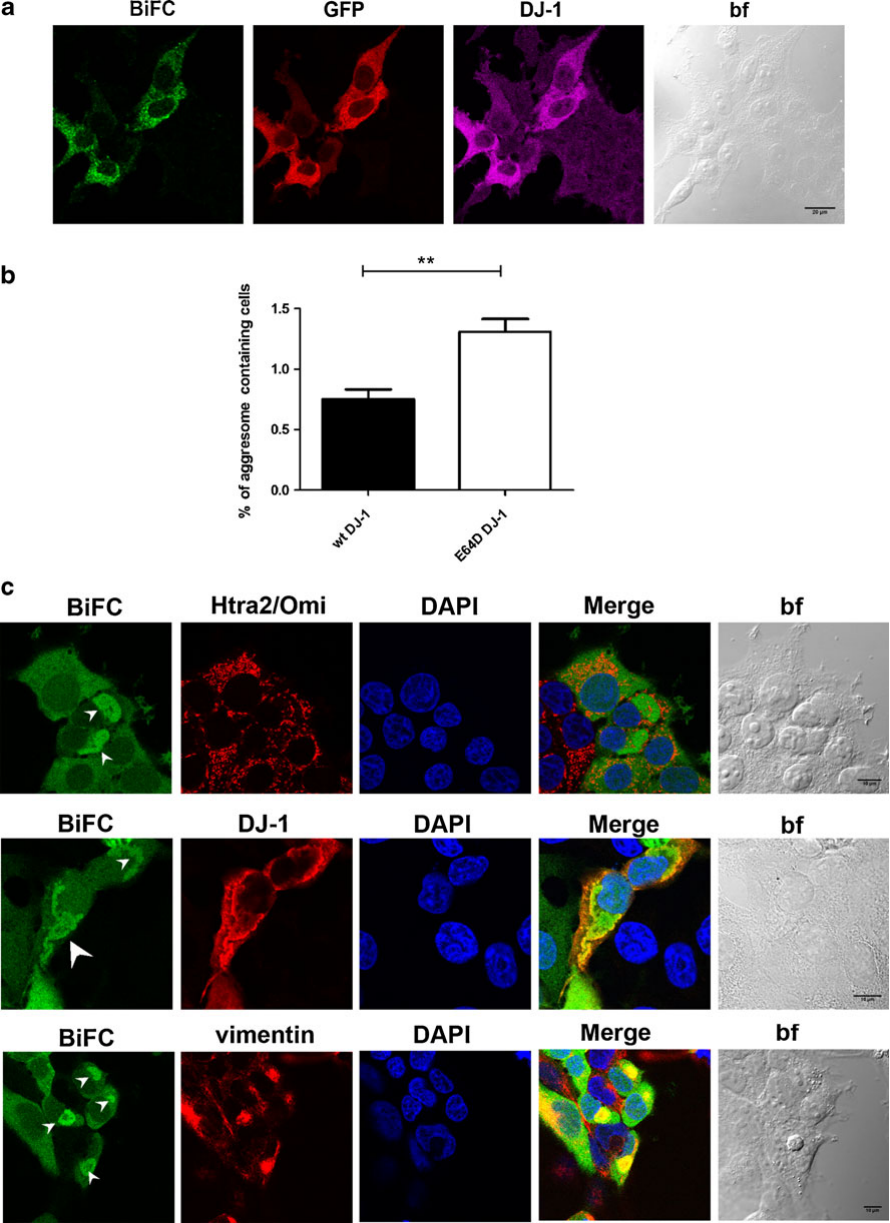
E64D DJ-1 forms cytoplasmic inclusions in living cells. **a** CLSM analysis of WT DJ-1 BiFC signal 24 h after transfection in HEK 293T cells after fixation and double labelling with both anti GFP and anti DJ-1 antibody. ICC experiments confirm the immunopositivity of WT DJ-1 transfected cells for both GFP and DJ-1. The anti DJ-1 antibody also detected endogenous DJ-1. Scale bar = 20 μm. **b** Percentage of WT and E64D cells containing aggresomes 48 h after transfection. **P < 0.01. **c** HEK 293T cells transfected with the two E64D DJ-1 BiFC constructs were fixed 48 h after transfection and subjected to immunostaining for the mitochondrial marker HtrA2/Omi, DJ-1, and the intermediate filament protein Vimentin. Scale bar = 10 μm

We next examined the dimerization signal obtained in cells transfected with WT and E64D DJ-1 by CLSM. Time course experiments revealed that BiFC complementation could be observed as early as 18 h posttransfection at 37 °C and that the brightest signal was obtained 36–48 h posttransfection for both WT and E64D DJ-1 constructs (data not shown). Analysis of the BiFC signal at 24 h posttransfection—the time point used for the BiFC experiments—showed that DJ-1 dimer localizes primarily to the cytoplasm, but is also found in the nucleus under control conditions (Fig. 6a). In agreement with the BiFC signal, immunocytochemistry (ICC) using GFP antibodies clearly labels transfected cells, while ICC using DJ-1 antibodies detected expression of DJ-1 in both transfected (brighter) and untransfected (weaker) cells, indicating the presence of endogenous DJ-1. No difference in subcellular localization was observed between DJ-1 dimer and endogenous DJ-1. The complementation signal 48 h after transfection was brighter for both WT and E64D BiFC constructs, and intracellular inclusion bodies containing DJ-1 dimers were detected in both WT and E64D DJ-1 transfected cells (Fig. S3). Provocatively, we found that the number of cells with inclusions was increased by ~75 % in cells expressing E64D DJ-1 versus WT DJ-1, indicating a specific effect of the E64D mutant on DJ-1 aggregation (Fig. 6b).

We then characterized these cytoplasmic inclusions by immunocytochemical studies and CLSM (Fig. 6c). We found that the E64D DJ-1 inclusions are recognized by an anti-DJ-1 antibody and have perinuclear localization. Furthermore, mitochondria are not localized within the inclusions, as shown by immunolabeling with the mitochondrial marker Htra2/Omi. Finally, we found that vimentin, an established marker for aggresomes, is recruited to the DJ-1 aggregates. Thus, these data suggest that E64D DJ-1 has an increased propensity to form aggresomes in living cells, which may have relevance to the mechanism(s) underlying pathogenesis of this mutation.

### E64D dimers show a distinct pattern of surface charge distribution

**Fig. 7:**
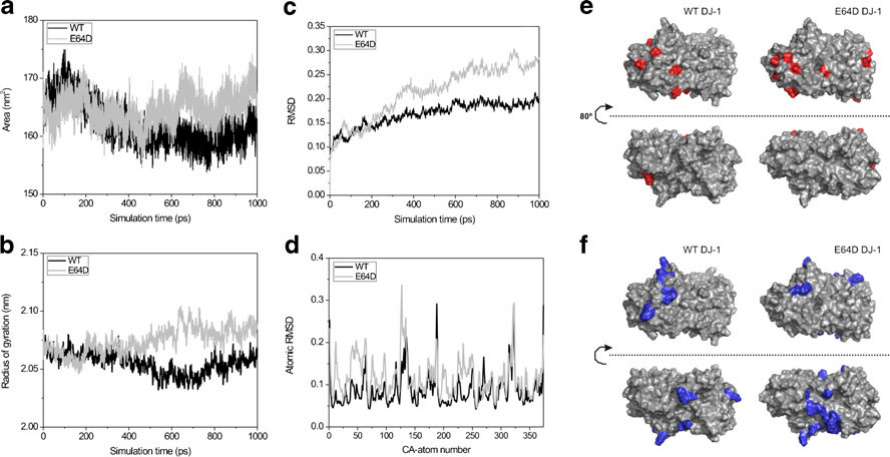
Analysis of the molecular dynamics trajectories obtained from the wild-type and E64D DJ-1 dimers during a 1,000-ps simulation in aqueous solution. **a** Exposed total surface area. **b** Radius of gyration. **c** Root mean square deviation values for the complete polypeptide chain along the simulation. **d** Root mean square deviation values for the atomic positions of C-alpha carbons in both species at the end of the simulation using as a reference the initial atomic coordinates in each model. **e**, **f** Representation of surface charged patches on the surface of WT and E64D DJ-1 dimer variants as calculated by Hotpatch software at the end of the MD simulation. **e** Negatively charged patches and **f** positively charged patches. Molecules are represented in two orientations obtained by a 180° equatorial turn

In order to further characterize the molecular properties of the E64D DJ-1 mutant, we performed molecular dynamics simulations (MD) of the dimeric protein variants in aqueous media within a time interval of 1,000 ps. Despite E64D localization at the surface of the molecule on the DJ-1 crystal structure, MD simulation data analysis clearly showed a strong influence of the mutation in the hydrodynamic properties of dimeric DJ-1 during the simulation (Fig. 7). Indeed, the total exposed surface area and the radius of gyration increased in the E64D mutant during the simulation in comparison with WT DJ-1 (Fig. 7a, b). Also evident are the molecular movements of the protein backbone as quantified by the global and atomic root mean square deviations (RMSD) (Fig. 7c, d). The E64D mutant showed an increased global RMSD value in the simulation, which demonstrated an increase in the vibrational entropy of the protein chain. Moreover, some regions within the protein backbone appeared to be more sensitive in E64D DJ-1, as calculated by the individual RMSD for C-alpha atoms (Fig. 7d). In particular, the E64D mutation leads to an increase in RMSD values for residues 30 to 60 in both of the monomeric components.

Interestingly, the observed molecular differences between WT and E64D DJ-1 were not restricted to the hydrodynamic properties of the proteins. Indeed, the distribution of electrostatic patches over the surface of both species at the end of MD simulation appears quite different (Fig. 7e, f). The The E64D mutant contains a highly positive exposed surface patch in the equatorial region of the dimer, which is not present in the wild-type protein (Fig. 7f). These observed differences in the hydrodynamic and electrostatic properties could suggest a basis for the differential behaviour of the mutant and wild-type proteins regarding aggregation propensity.

## Discussion

The determination of the DJ-1 protein structure [8, 9] first indicated that dimer formation was a stable and conserved oligomerization state for this protein. This has since been confirmed by biochemical approaches in vitro [4, 6, 10], suggesting that mutations responsible for dimer disruption cause a loss of function of DJ-1 activity. This hypothesis agrees with the recessive nature of DJ-1 associated PD, although to date DJ-1 protein function remains poorly understood. In the present study, we find that WT DJ-1 readily dimerizes in living cells, and this is completely prevented by the L166P mutation, in agreement with previous biochemical data [4, 10, 14, 16, 18], as well as structural and MD studies [19, 32]. Strikingly, using BiFC, we found that while the L166P DJ-1 mutant cannot dimerize with the WT protein, it can negatively impact the formation of WT DJ-1 dimers.

Despite the fact that both L166P and M26I mutations are localized to the dimer interface, previous studies investigating M26I DJ-1 dimerization are controversial. Some authors report M26I retains dimerization ability [4, 18, 33, 34], while other studies found decreased dimerization of the M26I mutant [20] and high protein destabilization resulting in a strongly reduced levels of M26I DJ-1 protein [35]. Our dimerization data support these latter results, which indicate that in living cells M26I mutant behaves like the L166P DJ-1 mutant. In our studies, this appears to be primarily due to a very low level of the M26I protein 24 h after transfection. In addition, our studies in living cells corroborate biochemical data on two more recently identified DJ-1 causative mutations—L10P and P158Δ [21]. Neither of these mutants is able to form homodimers, likely again as a consequence of the very low level of the mutant protein observed at the time of BiFC imaging.

A completely different picture has emerged for the E64D DJ-1 mutant based on previous in vitro studies, where normal dimerization has been observed by biochemical approaches [15, 19, 20]. In the present study, we were able to validate this result in living cells, finding no difference in dimerization between this mutant and WT protein. Despite normal dimerization, E64D DJ-1 has a decreased ability to prevent dopaminergic neurotoxicity elicited by different toxic stimuli in primary midbrain cultures [36], and human fibroblasts from homozygous carriers of the E64D mutation have reduced mitochondrial branching, a phenotype similar to that observed in murine embryonic fibroblasts from DJ-1 knockout mice [37]. Thus, understanding how the E64D mutation is causative in PD is a very intriguing aspect of DJ-1 biology.

Here, we provide evidence showing that the E64D dimer, despite retaining the capacity to dimerize, presents different phenotypic features compared to the WT protein. First, we found that, in living cells, the E64D dimer forms cytoplasmic inclusion bodies, and this is accompanied by a reorganization of the intermediate filament protein vimentin. This prompted us to use molecular dynamics simulations to verify whether new or different interactions are predicted to occur with the E64D dimer, which could explain the different aggregation propensity compared to WT DJ-1. Past studies have investigated the secondary and quaternary structure of the E64D DJ-1 [15, 20], but MD for the E64D dimer has not previously been performed. Our MD results indicate that, although the E64D mutation is localized far from the dimer interface, it has a strong effect on both the hydrodynamic and electrostatic properties of the dimer. Thus, either novel protein–protein interactions or protein instability caused by the altered surface charge distribution could be responsible for the formation of cytoplasmic E64D aggregates in control conditions. It is of interest to note that, although it is still unclear whether Lewy bodies are present in individuals with DJ-1 related forms of PD, nonfunctional aggregated DJ-1 is present in the brain of patients with neurodegenerative diseases [38], and DJ-1 levels are strongly increased in the detergent-insoluble fraction from sporadic PD and dementia with Lewy body brains [39]. Furthermore, DJ-1 forms aggregates in the presence of inorganic phosphate, levels of which are increased in PD patients [40]. Thus, our observations with E64D DJ-1 could have important pathogenic ramifications in PD.

We next used BiFC to interrogate the E64D DJ-1 dimer in oxidative stress conditions. The involvement of DJ-1 in oxidative stress response is well established in PD and is strictly linked to the maintenance of the healthy state of mitochondria within cells. For example, it is well known that, under oxidative stress conditions, the highly conserved cysteine residue at position 106 is oxidized to cystein-sulfinic acid [6], and this oxidation is critical for DJ-1 protection of mitochondria [4]. Our results clearly indicate that the E64D dimer does not react to oxidative stress in the same way as wild-type DJ-1. By testing two different oxidative stress stimuli, we observed that the WT DJ-1 dimer is stabilized in oxidative stress conditions, although this is not the case for the E64D DJ-1 dimer. We can thus hypothesize that structural/functional changes in E64D DJ-1 do not allow this mutant to respond normally in oxidative stress conditions. One possibility is that altered hydrodynamic/electrostatic properties predicted by MD for the E64D DJ-1 dimer may perturb its response to a redox switch. Further experiments will be required to elucidate the altered properties of E64D DJ-1 in control and oxidative stress conditions.

In summary, this work has provided new insights into both DJ-1 dimerization and the E64D DJ-1 mutation in living cells. We postulate that the E64D mutant, despite the fact that it appears to dimerize normally, has altered properties when compared to the WT protein. In addition, we have strongly validated BiFC as a tool for the study of DJ-1 dimerization and function. This approach will permit further analysis of DJ-1 function in living cells and provide a better understanding of the mechanisms underlying the pathogenesis of DJ-1 mutations, which cause familial PD.

## Electronic supplementary material

Below is the link to the electronic supplementary material.

ESM 1 (PDF 567 kb)
