# Supplementary material for: Parkinson’s disease-associated mutations in DJ-1 modulate its dimerization in living cells
Source: J Mol Med (Berl). 2012 Nov 27;91(5):599–611. doi: 10.1007/s00109-012-0976-y (PMC3644405; doi:10.1007/s00109-012-0976-y)
Supplement: Supplementary file 1 — (PDF 567 kb) [file 109_2012_976_MOESM1_ESM.pdf]

SUPPLEMENTARY MATERIAL

Parkinson’s disease-associated mutations in DJ-1 modulate its dimerization in living cells

Mariaelena Repici[1], Kornelis R. Straatman[2], Nadia Balduccio[1], Francisco J. Enguita[3], Tiago F. Outeiro[4,5,6†] and Flaviano Giorgini[1†]

[1]Department of Genetics, University of Leicester, UK;

[2]Centre for Core Biotechnology Services, University of Leicester, UK;

[3]Unidade de Biologia Celular, Instituto de Medicina Molecular, Lisboa, Portugal;
[4]Cell and Molecular Neuroscience Unit, Instituto de Medicina Molecular, Lisboa, Portugal;
[5]Instituto de Fisiologia, Faculdade de Medicina de Lisboa, Lisboa, Portugal;
[6]Department of Neurodegeneration and Restorative Research, University Medizin Göttingen, Göttingen, Germany

“J Mol Med 2012”

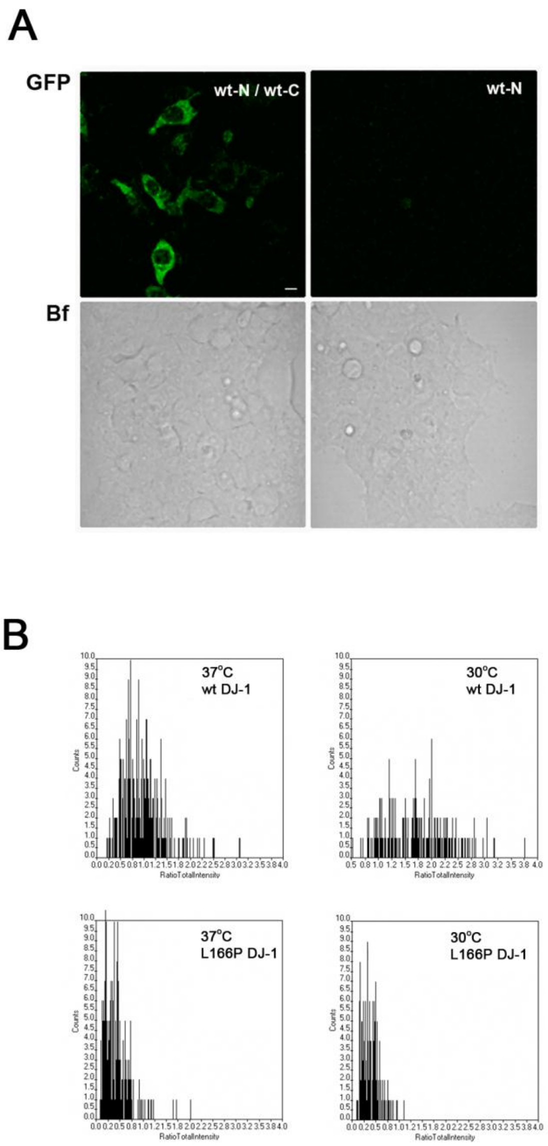

Figure S1. BiFC optimization studies. A) HEK293T cells were transfected with either one (WT DJ-1-GN173) or two BiFC constructs and fluorescence was observed 24 h after transfection. No signal was detected in cells transfected with only WT DJ-1-GN173. The same results were obtained for cells transfected with DJ1 –CC155 only (data not shown). Scale bar = 10 μm. B) The complementation signal obtained at 30 ºC for the two WT DJ-1 BiFC constructs was compared to the one obtained at 37 ºC. The distribution of the ratios clearly shows an enhanced effect of the lower temperature on the complementation signal, thus confirming that 30 ºC allows for a better maturation of the fluorophore.

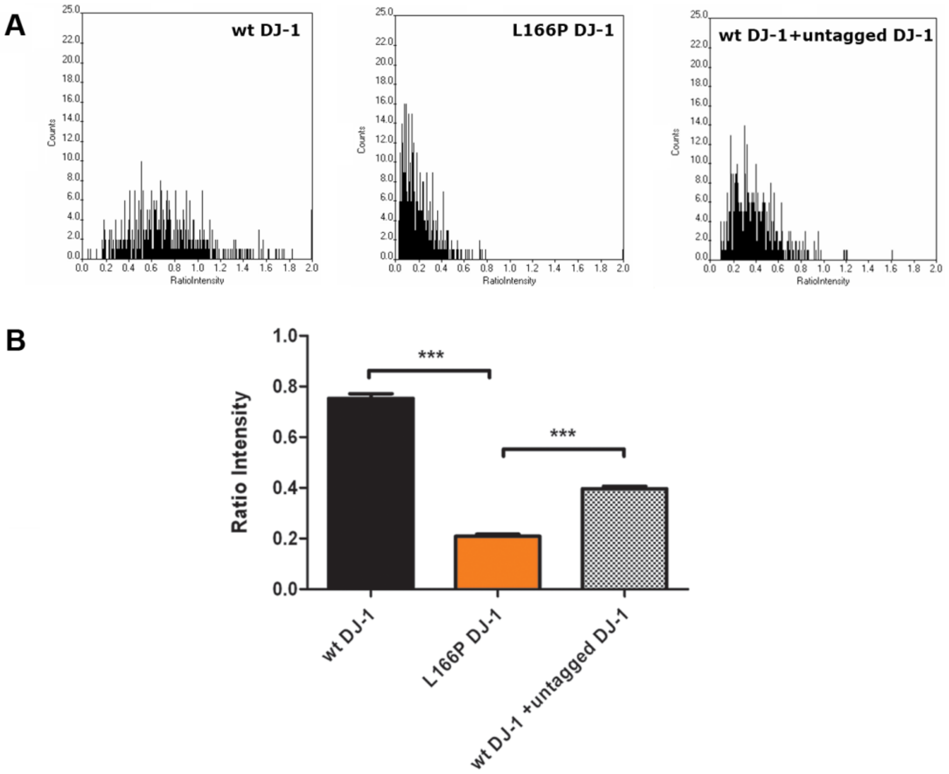

Figure S2. Effect of non-fluorescent DJ-1 on the efficiency of fluorescence complementation between DJ-1 BiFC constructs. A) HEK 293T cells were transfected with either the two WT DJ-1 BiFC constructs, the two L166P DJ-1 BiFC constructs or with the two WT DJ-1 BiFC constructs together with the "cold" non-fluorescent DJ-1 encoding plasmid (0.16 µg each). RFP encoding plasmid (0.08 µg) was used as an internal control. A decrease of ~47% in the complementation signal was observed when cold DJ-1 was used. B) The histogram shows the average ratio intensity (Green/Red) per well. *** P<0.001.

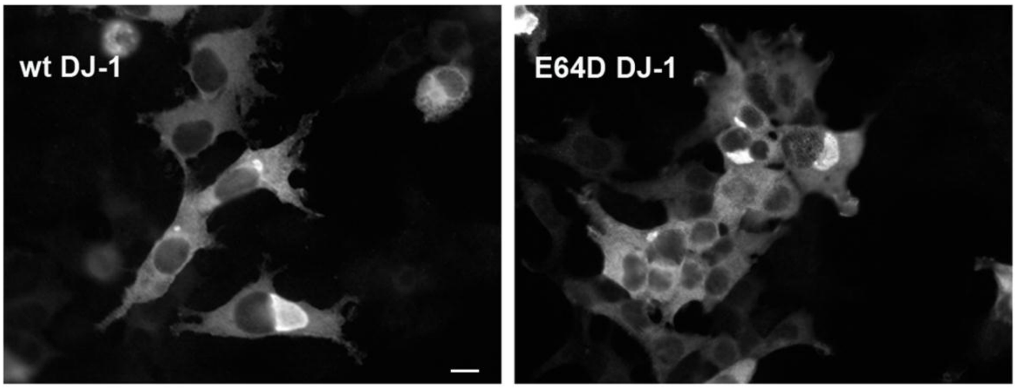

Figure S3. Representative images of WT and E64D DJ-1 inclusions 48 h after transfection of HEK 293T cells. Scale bar = 10 μm.

Primer Name	Sequence (5' to 3')
hDJ-1 L166P Fw	CAGCTTCGAGTTTGCGCCTGCAATTGTTGAAGCCC
hDJ-1 L166P Rev	GGGCTTCAACAATTGCAGGCGCAAACTCGAAGCTG
hDJ-1 M26I Fw	CATCCCTGTAGATGTCATTAGGCGAGCTGGGATTAAG
hDJ-1 M26I Rev	CTTAATCCCAGCTCGCCTAATGACATCTACAGGGATG
hDJ-1 E64D Fw	CTTGAAGATGCAAAAAAAGATGGACCATATGATGTGGTGG
hDJ-1 E64D Rev	CCACCACATCATATGGTCCATCTTTTTTTGCATCTTCAAG
hDJ-1 L10P Fw	GAGCTCTGGTCATCCCGGCTAAAGGAGCAG
hDJ-1 L10P Rev	CTGCTCCTTTAGCCGGGATGACCAGAGCTC
hDJ-1 P158Δ Fw	ACAAGCCGGGGGGGGACCAGCTTC
hDJ-1 P158Δ Rev	GAAGCTGGTCCCCCCCCGGCTTGT

Table S1. Primer pairs used for the generation of DJ-1 BiFC constructs by site directed mutagenesis.
